# Hospital utilization and out of pocket expenditure in public and private sectors under the universal government health insurance scheme in Chhattisgarh State, India: Lessons for universal health coverage

**DOI:** 10.1371/journal.pone.0187904

**Published:** 2017-11-17

**Authors:** Sulakshana Nandi, Helen Schneider, Priyanka Dixit

**Affiliations:** 1 Public Health Resource Network, India, Raipur, Chhattisgarh, India; 2 School of Public Health, University of the Western Cape, Bellville, South Africa; 3 School of Public Health, UWC/MRC Health Services to Systems Unit, University of the Western Cape, Bellville, South Africa; 4 School of Health Systems Studies (SHSS), Tata Institute of Social Sciences (TISS), Mumbai, India; Post Graduate Institute of Medical Education and Research School of Public Health, INDIA

## Abstract

Research on impact of publicly financed health insurance has paid relatively little attention to the nature of healthcare provision the schemes engage. India’s National Health Insurance Scheme or RSBY was made universal by Chhattisgarh State in 2012. In the State, public and private sectors provide hospital services in a context of extensive gender, social, economic and geographical inequities. This study examined enrolment, utilization (public and private) and out of pocket (OOP) expenditure for the insured and uninsured, in Chhattisgarh. The Chhattisgarh State Central sample (n = 6026 members) of the 2014 National Sample Survey (71^st^ Round) on Health was extracted and analyzed. Variables of enrolment, hospitalization, out of pocket (OOP) expenditure and catastrophic expenditure were descriptively analyzed. Multivariate analyses of factors associated with enrolment, hospitalization (by sector) and OOP expenditure were conducted, taking into account gender, socio-economic status, residence, type of facility and ailment. Insurance coverage was 38.8%. Rates of hospitalization were 33/1000 population among the insured and 29/1000 among the uninsured. Of those insured and hospitalized, 67.2% utilized the public sector. Women, rural residents, Scheduled Tribes and poorer groups were more likely to utilize the public sector for hospitalizations. Although the insured were less likely to incur out of pocket (OOP) expenditure, 95.1% of insured private sector users and 66.0% of insured public sector users, still incurred costs. Median OOP payments in the private sector were eight times those in the public sector. Of households with at least one member hospitalized, 35.5% experienced catastrophic health expenditures (>10% monthly household consumption expenditure).

The study finds that despite insurance coverage, the majority still incurred OOP expenditure. The public sector was nevertheless less expensive, and catered to the more vulnerable groups. It suggests the need to further examine the roles of public and private sectors in financial risk protection through government health insurance.

## Introduction

### Universal health coverage and government health insurance schemes

The concept of Universal Health Coverage (UHC) arose out of a global concern for high levels of out of pocket expenditure for health care in many low- and middle-income countries (LMIC) [1]. UHC has the goal of “ensuring that everyone within a country can access the health services they need, which should be of sufficient quality to be effective, and providing all with financial protection from the costs of using health services” [2: 3]. The three critical dimensions of UHC are: coverage of the population, coverage by services and financial protection [1]. Core to the design of UHC is the health financing system and how it engages with the mechanisms for provision of healthcare. Progress towards UHC requires strengthened health system functioning [1, 3–6] and a focus on equity [7–11].

Despite this broad vision, at country level, UHC has often focused on the establishment of state funded insurance schemes [12, 13] and stopped short of addressing the health systems strengthening or equity aspects of UHC. Kutzin [12: 607] raises this as a concern and calls for a shift in emphasis from a scheme to the health system in its entirety and for the “impact of that scheme on the attainment of the objectives for the population and system as a whole” to be monitored. The impact of state funded insurance schemes on financial protection and health equity are currently a subject of keen debate the world over, including in India [5, 13–16].

In their review of studies on the impact of national health insurance for the poor and the informal sector in LMICs, Acharya et al [17] found generally low enrolment rates in many of the schemes. There was lower enrolment among the poor, unless special efforts were made, and mixed findings with respect to rural versus urban enrolment. To date, gender has not been identified as a determinant of low enrolment [17–20]. Once enrolled, the evidence on subsequent utilization and financial risk protection is mixed [17], sometimes within the same country. While most studies found that insurance increased these parameters [21, 22], sometimes more for the poor [23], in others the impacts were unevenly distributed, with the poor benefitting less than the rich [17, 24–26].

The role of the health system capacity in determining the impact of the insurance schemes has been extensively documented [4, 20, 25–29]. However, few studies have explicitly disaggregated or compared the roles of the private and public sectors in achieving the objectives of UHC [1, 17].

Beyond the UHC debates, systematic reviews have concluded that the private health sector may not be more efficient than the public sector [30] nor result in greater access equity [28], although others maintain that the evidence is not conclusive [31].

### Government Health Insurance In India

India has a mixed health system, consisting of a network of government health facilities and health programmes as well as a dominant and unregulated private health sector [32, 33]. It is characterized by extensive inequities in health service utilization and access related to socio economic status, caste, geography, and gender, amongst others [33–39]. Private expenditure (including out of pocket payments) constitutes 70% of total health expenditure and 75% of primary and ambulatory healthcare episodes and 61% of inpatient episodes or hospital visits are in the private sector. Two percent (2%) of public sector expenditure relies on out of pocket fees and charges [32].

Studies of healthcare utilization patterns have found the poor in India are more likely to utilize the public sector for healthcare, making it pro-equity, than the private sector which relies predominantly on fee-for-service payment [39, 40]. However, Jain et al [16] argue that utilization of the public sector by the poor is not by choice but due to financial constraints that could be overcome by health insurance.

In India many players, including government, view state funded health insurance as the key mechanism to achieve UHC [41–44]. Over the last decades, states across India introduced government funded insurance schemes with the aim to protect the poor from catastrophic health expenditure [32, 45]. The National Health Insurance Scheme or RSBY, launched by the Ministry of Labour in 2007, and taken over by the Health Ministry in 2015, was the first national scheme for the unorganized sector, providing hospital cover for mainly Below Poverty Line (BPL) households. The key initial considerations for introducing RSBY were to promote India’s economic growth, the private healthcare market (drawing extensively on private healthcare providers) and worker productivity, especially in the informal sector [46, 47].

Health insurance schemes in India emerged in parallel to an existing major strand of health sector reform, namely the National Rural Health Mission (NRHM), launched in 2005, re-named the National Health Mission (NHM) in 2013, incorporating the Urban Health Mission. While the NHM aims to strengthen public health systems to provide “universal access to equitable, affordable and quality health care” [33, 48], the RSBY aims to actively draw in private sector providers through a “business model” involving both private and public sectors [49].

The stated objectives of RSBY are to provide financial protection and improve access to quality health care for the poor and other vulnerable groups, through “empowering the beneficiary” with “freedom of choice between public and private hospitals”, and providing “cashless” services [49].

The emerging evidence on the impact of the national and state government health insurance schemes in India shows that its beneficial effects have been, at best, limited. Some studies report an increase in enrolment and utilization of health care [50, 51, 52] and appropriate coverage of vulnerable groups [53]. However, in many others, socio-economic status, place of residence, caste, tribal group and women-headed households have emerged as significant determinants of inequity in enrolment and utilization [13, 37, 50, 54–58]. Moreover, instances of unnecessary hospitalizations and procedures and “provider-induced demand” have been documented, especially in the private sector [54, 59–65], also found in previous studies conducted by the authors in Chhattisgarh [66, 67].

Some studies have shown a decrease in out of pocket payments in those covered with insurance [52, 68, 69] but most find that enrolled patients continue to pay out of pocket, more so in the private sector, with instances of increased payment also reported [52, 58, 60, 65, 70–75].

### Government health insurance in chhattisgarh

Chhattisgarh State has a population of more than 25 million people [76], 79% of whom have been identified as poor, and requiring food security support [77]. With 44% of its geographical area under forests [78], the population is predominantly rural (77%) [76]. In a complex landscape of social groupings, “Scheduled Tribes” (indigenous groups) constitute 31% of the total population and “Scheduled Castes” a further 13% [76], both of whom are considered as marginalized and socially excluded groups relative to the others (“Other Backward Classes” and “Others”) [35].

Although Chhattisgarh has recorded improvements in health status since it was formed in 2000, it is still one of the low performing states in India [79].

Chhattisgarh was one of the first states to launch RSBY in 2009, expanding the scheme to all families living in the state in 2012 through the Mukhyamantri Swasthya Bima Yojana (MSBY) or Chief Minister’s Health Insurance Scheme. This move is seen as positive [80], as targeting in social programmes often leads to exclusion of the poor and disadvantaged [13, 81]. Both schemes (RSBY and MSBY) have identical provisions. They cover a family of five for pre and post hospitalization expenses up to an annual limit of Rs.30, 000 (US$ 442), with a one-time registration fee of Rs.30 to be paid by the family. Private and government hospitals are “empanelled” to provide services through pre-determined packages, reimbursed at fixed rates. As per the government data of April 2016, around 12.5 million people in Chhattisgarh are enrolled under RSBY/MSBY [82], mostly mobilized through processes involving rural grassroots workers, like the *Mitanins* (Community Health Workers) [83]. Of the 735 hospitals empanelled, 462 (62.9%) are private facilities [84]. Programme data for the 2015–16 financial year shows that the public sector made a smaller proportion of total number of claims (25.3%) than the private sector (74.7%), while the private sector received 82.9% of the claim amounts disbursed [84].

### Rationale for the study

Evidence on the extent of financial protection through government funded insurance schemes is mixed both globally and in India. Further, little attention has been given to evaluating the healthcare provision mechanisms government insurance schemes engage, and on the differential effects of public and private sector use on financial protection and reducing inequity. Moreover, recent debates related to measuring financial protection for UHC in the Sustainable Development Goals (SDGs) emphasize the need for looking at household expenditure on health and its ‘impoverishing effect’, instead of simply measuring coverage with an insurance scheme [85].

In Chhattisgarh, both public and private sectors are involved in providing services under the insurance scheme, in a context of extensive geographical, socio economic and gender inequities. The state funded Universal Health Insurance Scheme in Chhattisgarh provides the opportunity to study these elements and explore the pathways of utilization and extent of financial protection.

## Materials and methods

### Conceptual framework

**Fig 1:**
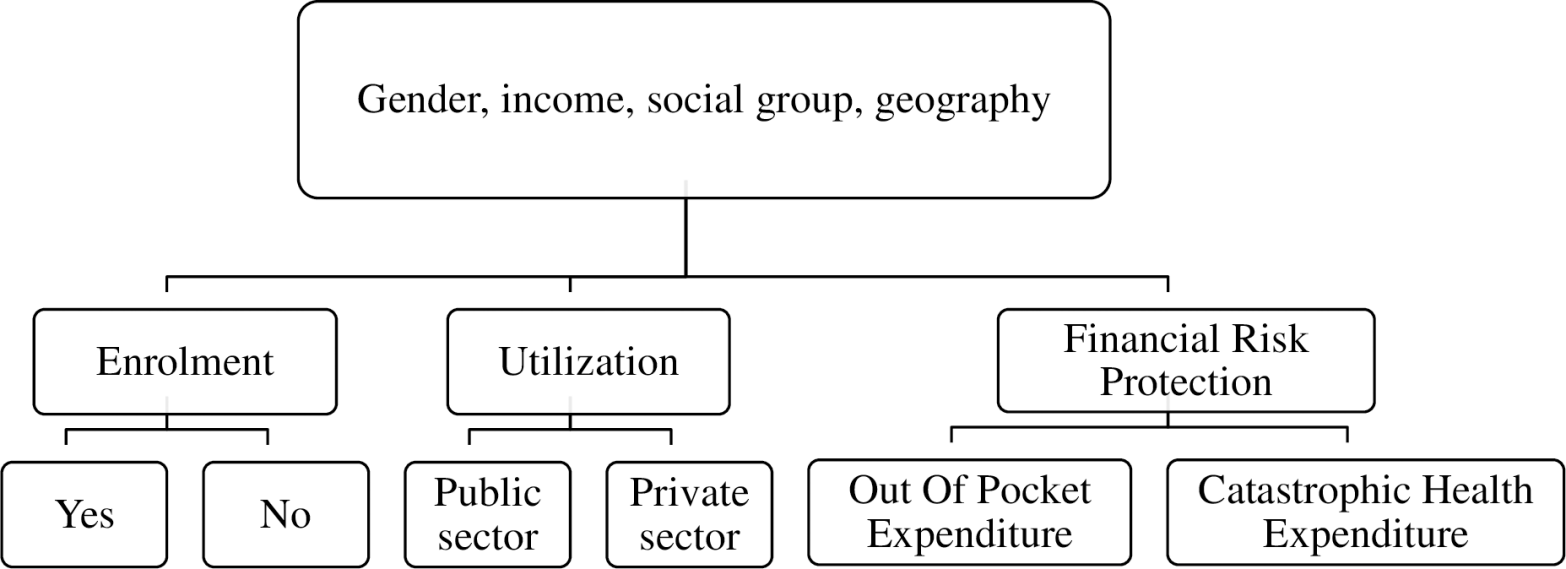
Conceptual framework for the study.

The conceptual framework for the study is illustrated in Fig 1. It represents the relationships between enrolment (yes/no), utilization (public and private sector hospitalization) and financial risk protection (out of pocket and catastrophic household expenditures).

### Study design, sampling and data collection

Drawing on household survey data, this descriptive study aimed to examine the relationships between enrolment, utilization of public and private sector sectors and financial risk protection for the insured and uninsured under the state funded health insurance in Chhattisgarh.

The Chhattisgarh State data used in this study were extracted from the 25th schedule of the 71st round of the cross-sectional Indian National Sample Survey, conducted between January and June 2014. The National Sample Survey Office (NSSO), under the Ministry of Statistics of the Government of India, conducts the survey on a periodic basis. The data is available from the Deputy Director General, Computer Centre, Ministry of Statistics and Programme Implementation, Government of India, New Delhi. The Chhattisgarh sample included 1205 households and 6026 individuals (household members), obtained in a stratified two-stage sampling design, with census villages and urban frame survey blocks as the first-stage units (FSUs) for the rural and urban areas respectively, and households as the second-stage units (SSUs).

The survey collected data in face-to-face interviews, using an interview schedule, on morbidity (self-reported), utilization of health care services (including types), and household expenditure on health care. Information was collected on every event of hospitalization of a household member, whether living or deceased at the time of survey, during the 365 days preceding the date of enquiry [86].

Information on household consumption expenditure was collected to create a consumption aggregate in the 30 days prior to the survey. Questions were asked to assess the “sum total of monetary values of all goods and services usually consumed (out of purchase or procured otherwise) by the household on domestic account during a month” [86: 8].

The NSSO survey does not ask about the specific type of government funded insurance scheme in its question on enrolment. The government health insurance schemes in the State, other than RSBY/MSBY are the Employees’ State Insurance Scheme (ESIS) and Central Government Health Scheme (CGHS). However, coverage data of these schemes reveal that during the period of the study the families covered under RSBY/MSBY made up the highest proportion of the enrolled under any government insurance. Under the RSBY 4^th^ round of enrolment for 2013–2015, the number of enrolled families was 38,28,024 [87]. Under the Employees’ State Insurance (ESI) in 2014, 2,50,720 families were covered [88]. In 2014 (year of the NSSO survey), RSBY/MSBY thus constituted 93.9% of the enrolled. The Central Government Health Scheme (CGHS) gives coverage to central government employees residing in ‘CGHS-covered cities’, however, no areas or cities in Chhattisgarh are designated as CGHS-covered cities [89]. Moreover, CGHS eligibility [89] also includes retired central government personnel, of whom the numbers residing in Chhattisgarh would be very small. Hence the data on insured in government insurance schemes in the NSSO survey primarily reflects the coverage under RSBY/MSBY.

### Analysis

The NSSO used a multistage sampling design that is not self-weighting. The NSSO provides the appropriate weights for analyses to ensure representativeness of aggregated data. These were applied in all the analyses, unless otherwise specified. The details of the sampling weights, methods and organization of the NSSO are reported elsewhere [86].

Descriptive analyses of the elements in Fig 1 (enrolment, hospitalization, use of public and private sectors, out of pocket and catastrophic expenditures) were conducted. The usual monthly per capita consumer expenditure (UMPCE) was calculated as the household’s usual consumption expenditure in a month divided by the size of the household and then divided into five economic quintiles, from Q1 (poorest) to Q5 (richest). Out of pocket expenditure on hospitalization was calculated per episode as medical expenditure minus reimbursements. Weighted medians of OOP expenditure were calculated. The methodology proposed by Wagstaff and van Doorslaer [90] was applied for assessing catastrophic payments for health care, namely, expenditure that exceeded 10% of annual total household consumption expenditure.

Further, multivariate logistic analyses were undertaken to examine the following relationships:

- Between enrolment and variables of gender (women-men), social group (Scheduled Caste, Scheduled Tribe, Other Backward Classes and General), place of residence (urban-rural) and UMPCE (referred to collectively as socio-economic factors).
- Between hospitalization and the above socio-economic factors, adding enrolment status.
- Between public sector hospitalization and the above socio-economic factors, adding enrolment and type of ailment.
- Between OOP expenditure and socio-economic factors, enrolment, type of ailment and level of facility.

Adjusted Odds Ratios (AOR) and 95% confidence intervals (CI) were estimated for each of the models.

In the logistic regressions, outcome variable was coded as ‘1’ for an individual enrolled in government insurance scheme and ‘0’ for an individual not enrolled in any insurance scheme; ‘1’ for an individual who was hospitalized and ‘0’ if not; ‘1’ if an individual was hospitalized in the public sector during last 365 days from the date of survey and ‘0’ if hospitalized in the private sector; ‘1’ if incurred any OOP expenditure and ‘0’ if did not incur any OOP expenditure. The binary response (‘y’), enrolled in government insurance scheme or not/hospitalized or not/hospitalized in public sector or private sector/incurred OOP expenditure or not) for each individual was related to a set of categorical predictors, ‘X’, and a fixed effect by a logit link function as follows:

$$logit\;(\pi_{i})=\log[\pi_{i}/1-\pi_{i}]=\beta_{0}+\beta (x)+\varepsilon$$

The probability of an individual who had enrolled in government insurance scheme/hospitalized/hospitalized in public sector/incurred OOP expenses is *π*_*i*_. The parameter *β*_0_ estimates the log odds of enrolled in government insurance scheme/hospitalized/ hospitalized in public sector/incurred OOP expenses for the reference group, and the parameter *β* estimates with maximum likelihood, the differential log odds of enrolled in government insurance scheme/hospitalized/ hospitalized in public sector/incurred OOP expenses are associated with the predictor X, as compared to the reference group and ε represents the error term in the model.

As the data is from an official government survey, the researchers did not have any control over the validity or reliability of data. However, it is regarded as one of the most important sources of public health data in India, having high validity [91].

### Ethics approval

The Senate Research Committee of the University of the Western Cape gave ethics approval for this secondary analysis, as part of the PhD studies of the first author.

## Results

### Characteristics of the study sample

The gender, residential (urban/rural) and social group distribution (number and weighted percentage), of the 6026 household members, is shown in Table 1. The study used the complete sample in its analysis.

**Table 1:** Characteristics of the study sample (N = 6026).

Characteristic		N	W %
**Gender**	Men	3,080	53.4
	Women	2,946	46.6
**Residence**	Rural	3,524	81.9
	Urban	2,502	18.1
**Social Group**	ST	1,895	34.6
	SC	655	12.6
	OBC	2,694	45.8
	Others	782	7.0

### Coverage with insurance

Of the total surveyed, 38.8% were covered by any government insurance scheme, which includes both the universal insurance scheme and central and state schemes for government employees (Table 2). A further 0.5% was covered with private insurance, while 60.7% of the sample had no insurance coverage of any kind.

**Table 2:** Enrolment in insurance by different characteristics and results of adjusted odds ratio of insurance enrolment and its 95% CI (N = 5977)*.

Characteristic		N	Total	Enrolled in insurance	Not enrolled in any insurance	Adjusted Odds Ratio	P value	95% Confidence Interval	
			w%	w %	w %			Lower limit	Upper limit
**Total**		5,977		38.8	60.7				
**Gender**	Men^#^	3,055	53.4	52.6	54.0	1			
	Women	2,922	46.6	47.4	46.1	0.919	0.120	0.828	1.022
**Residence**	Rural^#^	3,506	81.9	83.9	80.6	1			
	Urban	2,471	18.1	16.1	19.4	0.885	0.063	0.786	1.013
**Social Group**	ST^#^	1,875	34.4	36.3	33.2	1			
	SC	649	12.6	11.9	13.1	0.750	0.006	0.642	0.928
	OBC	2,680	46.0	46.2	45.8	0.634	0.000	0.561	0.719
	Others	773	7.0	5.6	7.9	0.416	0.000	0.342	0.516
**UMPCE**	Q1^#^	1,203	24.9	24.2	25.3	1			
	Q2	1,199	24.3	25.9	23.3	0.840	0.031	0.701	0.973
	Q3	1,189	21.4	24.1	19.7	1.093	0.287	0.929	1.291
	Q4	1,205	18.9	20.2	18.0	1.184	0.049	1.004	1.404
	Q5	1,181	10.6	5.6	13.7	0.654	0.000	0.516	0.761

Note

* 49 individuals had private insurance and therefore are not included in this analysis

# Reference category

Henceforth, ‘insurance’ refers only to government health insurance, and no further data on private insurance is presented.

Table 2 gives the socio-economic characteristics of the insured and uninsured. When gender, residence, social group and consumption expenditure (UMPCE) were combined in a logistic regression model, with enrolment as an outcome variable (Table 2), social group and UMPCE emerged as predictors of coverage. Scheduled Tribes were significantly more likely to be enrolled than other social groups while the richest (Q5) were significantly less likely to be enrolled (AOR 0.654; 95% CI: 0.516–0.761) among the UMPCE groups.

### Hospitalization and choice of facility

Of the sample, 817 persons were hospitalized during the prior 365 days, with a total of 856 episodes of hospitalization. Weighted rates of hospitalization were 33 per 1000 in those with insurance, compared to 29 per 1000 in those with no insurance. After controlling for gender, place of residence, social group and UMPCE quintile, a person with insurance was significantly more likely to be hospitalized compared to a person with no insurance (AOR 1.388; 95% CI: 1.190–1.620) (S1 Table).

**Fig 2:**
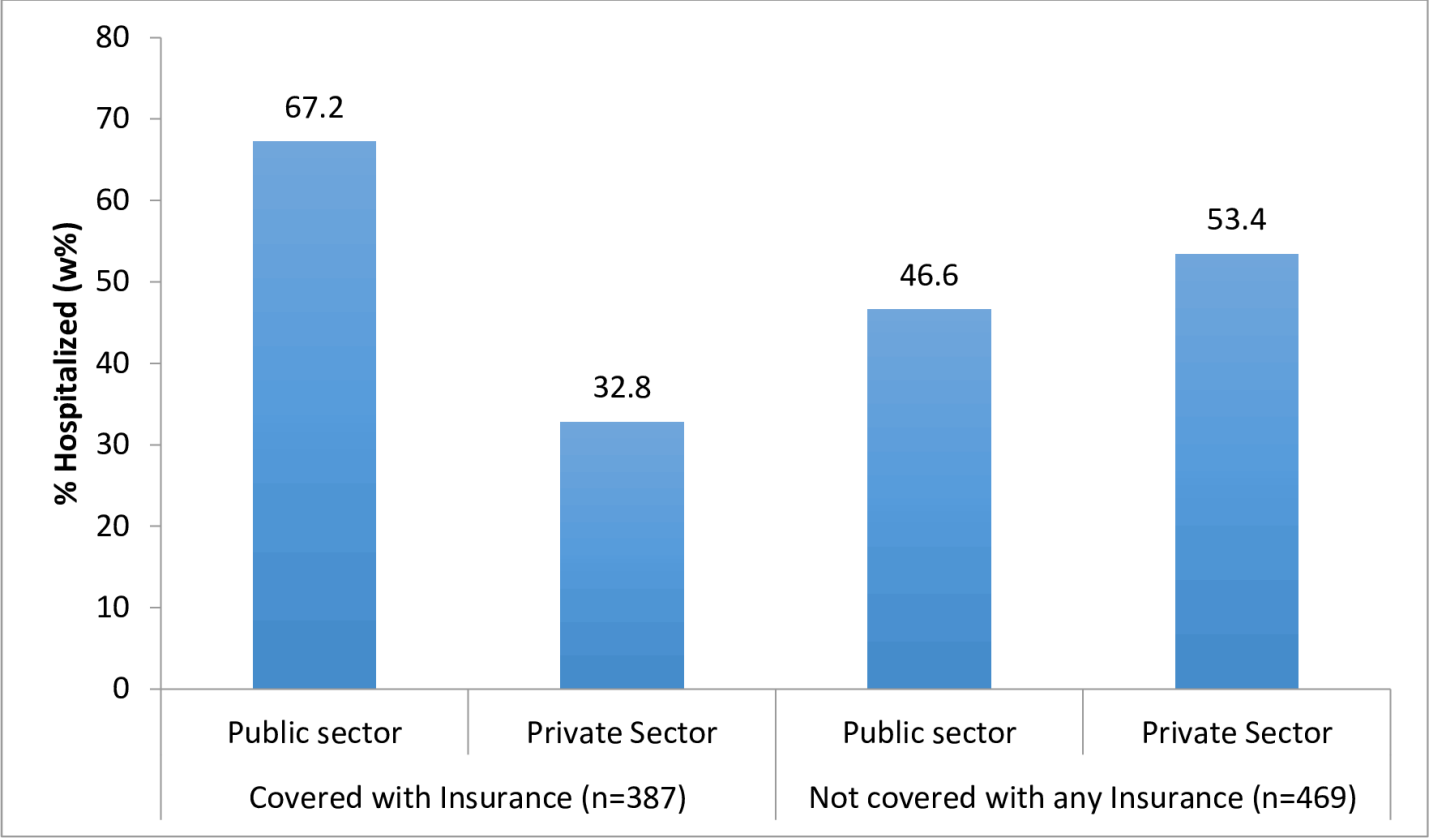
Proportion of hospitalization by in public and private sector by insurance coverage (n = 856).

In those covered by insurance, two thirds of hospitalization episodes were in the public sector (67.2%), compared to less than half (46.6%) in those with no insurance (Fig 2).

The level of facility for hospitalizations for the insured and uninsured are given in Table 3. It shows that most of the hospitalizations were in the higher level facilities both in the public and private sectors, which for the public sector means that they were in district hospitals and medical colleges (as opposed to lower level health centers).

**Table 3:** Place of hospitalization by insured and uninsured (n = 856).

Level of facility	Enrolled in insurance		Not enrolled in any insurance		Total	
	N	W %	N	W %	N	W %
Sub center/ASHA/AWW	5	3.2	7	2.5	12	2.8
PHC/Dispensary/CHC/Mobile medical unit	27	5.7	23	5.4	50	5.5
Public hospital	195	58.4	190	38.7	385	47.0
Private doctor/clinic+	-	-	-	-	-	-
Private hospital	160	32.8	249	53.4	409	44.7
Total	387	100	469	100	856	100

+ No cases found hospitalized under this category

The multivariate logistic regression showed that women (AOR 1.80; 95% CI: 1.25–2.58), Scheduled Tribes and the poorest (Q1) were significantly more likely to be hospitalized in the public sector than men, other social groups and other UMPCE groups respectively (S2 Table). Taking infection as the reference group, conditions like cancer (AOR 0.11; 95% CI: 0.01–0.94) and respiratory conditions (AOR 0.30; 95% CI: 0.09–0.97) were significantly less likely causes of admission in the public sector, while obstetric and child birth-related conditions were significantly more likely in the public sector (AOR 1.63; 95% CI: 1.03–2.57) (S2 Table). Enrolment in government insurance was associated with hospitalization in the public sector at 90% Confidence Levels (AOR 1.32; 90% CI: 1.01–1.72) (S2 Table).

### Out of pocket expenditure

Of those with insurance, 34.0% of hospitalization episodes in the public sector were ‘cashless’, that is, no OOP expenditure was incurred, whereas 16.1% of public sector users without insurance got cashless services. For those going to the private sector, 5.0% of the insured and 5.7% of those not insured did not incur any OOP expenditure. In those with insurance who incurred OOP expenditure, the median OOP expenditure in private (Rs.10, 000) was eight times more than in the public sector (Rs.1, 200). In the uninsured, median OOP expenditure in private (Rs.17, 900) was nearly twelve times higher than in the public sector (Rs.1, 500).

Table 4 gives the median OOP expenditure disaggregated by insurance coverage and socio-economic categories, although analysis is limited by small sample sizes in the disaggregated analysis precluding public/private comparisons.

**Table 4:** Median OOP expenditure (OOPE) (medical expenses minus reimbursements) per hospitalization episode for various categories (N = 856).

Characteristic		Enrolled in insurance		Not enrolled in any insurance	
		N	Median OOPE (Rs.)	N	Median OOPE (Rs.)
**Total**		387	2550	469	4500
**Gender**	Male	167	2500	180	6400
	Female	220	3080	289	3000
**Residence**	Rural	230	2500	208	3370
	Urban	157	5900	261	6000
**Social Group**	ST	137	2500	89	1550
	SC	52	5000	55	3500
	OBC	162	5500	224	6400
	Others	36	2000	101	9900
**UMPCE**	Q1	70	1200	73	2000
	Q2	57	0	76	2200
	Q3	94	2500	86	3000
	Q4	95	4200	84	6400
	Q5	71	10000	150	27000

Multivariate logistic regression with OOP expenditure (Y/N) as the outcome variable showed that government insurance coverage (AOR 0.265; 95% CI: 0.174–0.405) and childbirth conditions (AOR 0.516; 95% CI: 0.290–0.918) were significantly less likely to entail OOP expenditure than no insurance and other ailments respectively (S3 Table). On the other hand, women (AOR 1.700; 95% CI: 1.012–2.858) were more likely to incur OOP expenditure than men and hospitalization in private hospital had a significantly higher possibility of incurring OOP expenditure than any other type of facility (S3 Table).

**Fig 3:**
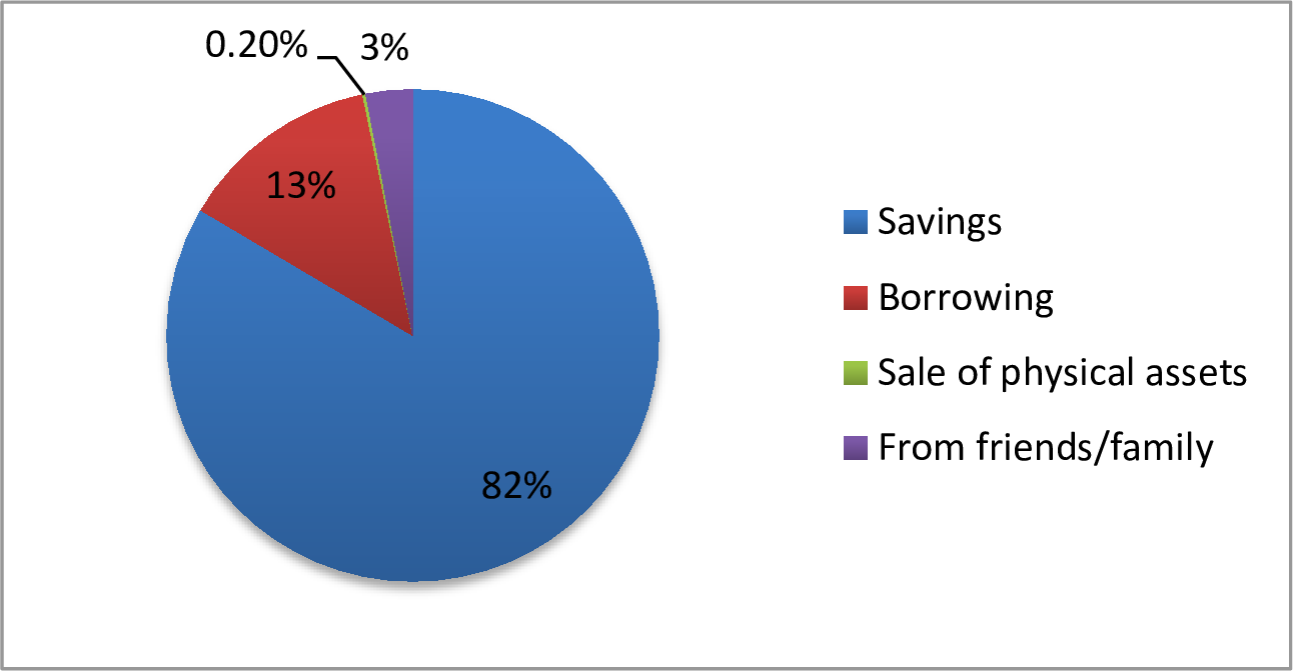
Source of funds for OOP expenditure.

Among people who were hospitalized and incurred OOP payments, 82% used their savings, and 13% borrowed money (Fig 3). The others took money from friends or family (3%), sold physical assets (0.2%) or arranged for it in some other way (2%).

### Catastrophic expenditure due to hospitalization costs

Household catastrophic expenditure due to hospitalization was calculated for the 645 households where at least one person was hospitalized during the prior 365 days. Using 10% of household consumer expenditure on OOP expenditure for hospitalization as the cut-off mark, 35.5% of the households experienced catastrophic expenditure due to hospitalization costs. It was not possible to assess the effects of insurance coverage on this as within the households, members had a mixed profile of enrolment.

**Fig 4:**
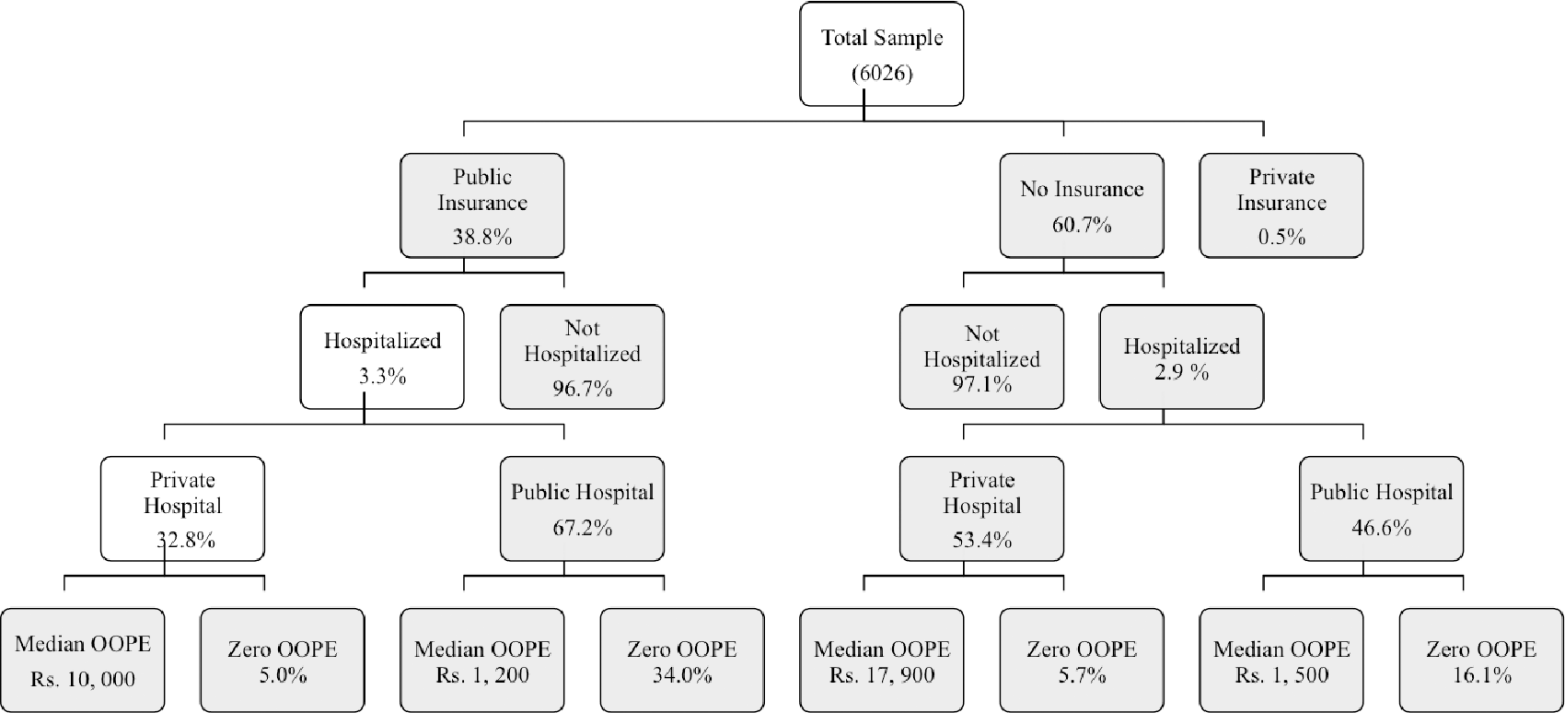
Summary of study findings.

Fig 4 summarizes the main findings on the study dimensions, based on the conceptual framework (Fig 1).

## Discussion

This study, using the National Sample Survey (71^st^ Round) on Health conducted in 2014, explored coverage of government insurance schemes, utilization of hospitalization and out of pocket expenditure for the insured and uninsured in Chhattisgarh State of India. The discussion below examines these findings in the context of other studies and their relevance to the larger debates on government insurance and UHC.

Chhattisgarh, a state with predominantly rural and poor populations started implementing RSBY in 2009 and universalized the scheme in 2012. At the time of the study (2014) enrolment percentages were low, although the recent programme data shows continuing growth—the number of families enrolled increased from 1.04 million in March 2011 (before universalization) [72] to 4.16 million in April 2016 [82]. The study found that enrolment was marginally higher in rural areas, among women and Scheduled Tribes, compared to the total covered. These finding on gender and rural residence echo findings from other studies [17, 18, 52].

The rates of hospitalization for those who were covered with insurance were slightly higher than for those not covered with insurance. The evidence on impacts of insurance on utilization elsewhere is mixed [17]. In the utilization of hospital services, one of the critical purposes of the health insurance scheme is to “empower” people by providing freedom of choice to go to a public or private sector facility [49]. Jain et al [16] argue that a purchasing mechanism like health insurance can make the private sector more accessible to the poor. However, this study shows that even when insured, people appear to be utilizing the public sector more. Certain explanations could be drawn using the evidence from this and other studies. Firstly, multivariate logistic regression on public sector hospitalization shows that women, tribal populations and poorest are significantly more likely to go to the public sector. Other studies too have documented the higher use of the public sector by poorer populations [40] and the lower availability of private facilities in poorer and rural areas [36, 39, 40]. Moreover, a recent study comparing two rounds of NSSO data for whole of the country has found that use of public sector hospitals has increased and for the insured, there is higher probability of being hospitalized in a public, rather than a private hospital [92]. Secondly, our study also shows obstetrics and gynecological conditions were significantly more likely to be hospitalized in the public sector. The National Family Health Survey-4 data of Chhattisgarh shows that deliveries in the public sector increased by eight times over ten years (from 6.9% in 2005–06 to 55.9% in 2015–16), one of the highest increases in the country [93]. Therefore the high number of public sector hospitalizations could be related to the high public sector utilization by women for delivery and other conditions, which has also emerged from other studies [92]. Thirdly, the data on OOP expenditure shows that there was greater probability of incurring expenditure in the private sector and the median amounts in the private sector even for the insured were higher than in the public sector. Lack of financial protection is a critical barrier to access and utilizing health services [1, 2] and therefore higher affordability of the public sector may have led to more people utilizing it.

Although the study shows a higher rate of utilization of the public sector by the people who were enrolled in the government insurance, programme data of the scheme shows that the insurance card is being used more in private than in public sector [84]. One possible explanation for this difference could be that although people are making greater use of the public sector, they may not be routinely using the insurance card in the public sector.

Financial protection has been the mainstay of any government insurance scheme. For RSBY too, providing “cashless” hospitalization services and reducing catastrophic expenditure for hospitalization has been highlighted as the most important objective of the scheme [87]. The results of the study show that although the insured were less likely to incur OOP expenditure than the uninsured, most of the insured had to incur OOP expenditure. One third (35.5%) of the households experienced catastrophic health expenditure (CHE) due to medical expenses for hospitalizations.

Studies both from India [52, 68, 69] and other countries [21, 22] have found evidence of financial protection from insurance schemes. However, most studies from India also show that patients continue to incur OOP expenditure despite coverage with government insurance [15, 58, 60, 65, 70, 71, 73, 75]. A recent systematic review on the impact of publicly financed health insurance schemes found that though utilization increased with coverage, there was no impact on reduction of OOP expenditure [51]. Moreover, studies have also shown that the impact is often less on the poor and rural populations [17, 25, 51, 68, 75]. Analyzing the same NSSO survey data for the whole of India, Sundararaman et al [15] argue that the difference in net OOP expenditure between the insured and uninsured is too small to claim financial protection.

Comparing OOP expenditure in the public and private sectors, “cashless” hospitalizations were more common in public than in private facilities and those going to the private sector were more likely to incur OOP expenditure. Where OOP expenditure was incurred, amounts were eight times higher in private than in public facilities for people covered with insurance. Previous work in Chhattisgarh [72, 94] by the authors, and by Rent & Ghosh [75] in neighbouring Maharashtra has found similar differences. It is pertinent to note that in Chhattisgarh, the private sector receives the major share (82.9%) of claim amounts, and accounts for two-third of hospitalizations under the scheme [82]. While the NSSO data-set does not indicate whether the insurance was actually used during hospitalization, it is assumed that those covered would try to utilize it and ensure “cashless” hospitalizations. The findings of this analysis suggest that the core RSBY/MSBY goal of “cashless” utilization of health facilities is far from being achieved.

## Limitations

The NSSO survey data on enrolment includes enrolment in ESIS and CGHS in addition to RSBY/MSBY, although, as discussed in the methods, the RSBY/MSBY made up the highest proportion of the enrolled under any government insurance.

The study found that in the private sector, 5.7% of the uninsured did not incur OOP expenditure. On examining these six cases, no pattern was found in terms of their socio-economic characteristics, age, rural/urban residence or type of ailment, and no reason could be gauged for the zero OOP expenditure. There may also have been a problem of recall bias in these cases.

Chhattisgarh is a predominantly rural state, as is the case with most of India. Of India’s population, 69% is rural with more than half (16 out of 29) of the states having rural populations of above 70% [95]. All states have a similar healthcare system, with a private/public mix, and with government insurance schemes primarily relying on private providers. However, in most states, the insurance scheme has not been universalized and enrolment in the schemes is much lower than in Chhattisgarh. Nevertheless, universal insurance coverage is seen as a move towards UHC [13]. Therefore the findings on enrolment, private and public sector utilization, and OOP expenditure for the insured and uninsured, which emerge from this study, in the context of geographical, socio-economic and gender inequities, are relevant for India and have lessons for UHC elsewhere. It also illustrates the relevance of the recently changed indicator for measuring financial risk protection of UHC in the SDGs [96].

## Conclusion

This study of Chhattisgarh’s universal government health insurance scheme found that despite insurance coverage, most had to incur OOP expenditure, which was higher in the private than the public sector. Moreover, a large proportion of households with members hospitalized experienced catastrophic health expenditure. Whether through choice or availability, those with insurance coverage made greater use of services in the public sector. The public sector was less expensive, and catered to the more vulnerable groups. The patterns of utilization and differential OOP expenditure across public and private sectors under publicly financed health insurance warrant further investigation, so as to inform strategies that make best use of scarce public resources and deliver on the promise of equity under Universal Health Coverage.

## Supporting information

S1 Table Adjusted Odds Ratio of hospitalization by characteristics and its 95% CI (N = 5977). (DOCX)

S2 Table Adjusted Odds Ratio of hospitalization in the public sector by characteristics and its 95% CI (N = 856*). (DOCX)

S3 Table Adjusted Odds Ratio of OOPE (medical expenses minus reimbursements) by characteristics and its 95% CI (N = 856*). (DOCX)
