# Supplementary material for: Hospital utilization and out of pocket expenditure in public and private sectors under the universal government health insurance scheme in Chhattisgarh State, India: Lessons for universal health coverage
Source: PLoS One. 2017 Nov 17;12(11):e0187904. doi: 10.1371/journal.pone.0187904 (PMC5693461; doi:10.1371/journal.pone.0187904)
Supplement: S1 Table — (DOCX) [file pone.0187904.s001.docx]

**S1 Table: Adjusted Odds Ratio of hospitalization by characteristics and its 95% CI (N=5977*)**

| **Characteristic** | | **Adjusted Odds Ratio** | **P value** | **95 % Confidence Interval** | |
| --- | --- | --- | --- | --- | --- |
|  |  |  |  | **Lower Limit** | **Upper Limit** |
| **Gender** | Men# |  | 1 |  |  |
|  | Women | 1.686 | 0.000 | 1.449 | 1.963 |
| **Residence** | Rural# |  | 1 |  |  |
|  | Urban | 1.150 | 0.131 | 0.959 | 1.378 |
| **Social Group** | ST# |  | 1 |  |  |
|  | SC | 1.294 | 0.055 | 0.994 | 1.684 |
|  | OBC | 1.154 | 0.136 | 0.956 | 1.393 |
|  | Others | 1.285 | 0.068 | 0.982 | 1.681 |
| **UMPCE** | Q1# |  | 1 |  |  |
|  | Q2 | 0.916 | 0.502 | 0.708 | 1.184 |
|  | Q3 | 1.203 | 0.140 | 0.941 | 1.538 |
|  | Q4 | 1.149 | 0.281 | 0.893 | 1.478 |
|  | Q5 | 1.485 | 0.004 | 1.138 | 1.937 |
| **Insurance** | No Insurance# |  | 1 |  |  |
|  | Government Insurance | 1.388 | 0.000 | 1.190 | 1.620 |

Note: *49 individuals had private insurance and therefore are not included in this analysis. # Reference category
