# Supplementary material for: Hospital utilization and out of pocket expenditure in public and private sectors under the universal government health insurance scheme in Chhattisgarh State, India: Lessons for universal health coverage
Source: PLoS One. 2017 Nov 17;12(11):e0187904. doi: 10.1371/journal.pone.0187904 (PMC5693461; doi:10.1371/journal.pone.0187904)
Supplement: S2 Table — (DOCX) [file pone.0187904.s002.docx]

**S2 Table: Adjusted Odds Ratio of hospitalization in the public sector by characteristics and its 95% CI (N=856*)**

| **Characteristic** | | **Adjusted Odds Ratio** | **P value** | **95 % Confidence Interval** | |
| --- | --- | --- | --- | --- | --- |
|  |  |  |  | **Lower Limit** | **Upper Limit** |
| Gender | Men# | 1 |  |  |  |
|  | Women | 1.80 | 0.00 | 1.25 | 2.58 |
| Residence | Rural# | 1 |  |  |  |
|  | Urban | 0.89 | 0.54 | 0.63 | 1.28 |
| Social Group | ST# | 1 |  |  |  |
|  | SC | 0.40 | 0.00 | 0.23 | 0.68 |
|  | OBC | 0.28 | 0.00 | 0.19 | 0.42 |
|  | Others | 0.30 | 0.00 | 0.17 | 0.53 |
| UMPCE | Q1# | 1 |  |  |  |
|  | Q2 | 0.78 | 0.38 | 0.45 | 1.36 |
|  | Q3 | 0.99 | 0.96 | 0.58 | 1.67 |
|  | Q4 | 0.53 | 0.02 | 0.31 | 0.89 |
|  | Q5 | 0.26 | 0.00 | 0.15 | 0.46 |
| Insurance | No Insurance# | 1 |  |  |  |
|  | Government Insurance* | 1.32 | 0.08 | 0.96 | 1.81 |
| Type of Ailment | Infection# | 1 |  |  |  |
|  | Cancers | 0.11 | 0.04 | 0.01 | 0.94 |
|  | Blood Diseases+ Endocrine Metabolic, Nutritional | 0.91 | 0.80 | 0.43 | 1.91 |
|  | Psychiatric & Neurological | 0.52 | 0.11 | 0.23 | 1.15 |
|  | Eye | 0.65 | 0.30 | 0.29 | 1.46 |
|  | Cardio-Vascular | 1.20 | 0.68 | 0.51 | 2.79 |
|  | Respiratory | 0.30 | 0.04 | 0.09 | 0.97 |
|  | Gastro-Intestinal | 0.48 | 0.02 | 0.26 | 0.89 |
|  | Musculo-Skeletal+ Genito-Urinary | 0.34 | 0.00 | 0.17 | 0.67 |
|  | Obstetric+ Childbirth | 1.63 | 0.04 | 1.03 | 2.57 |
|  | Injuries | 1.21 | 0.51 | 0.69 | 2.12 |
|  | Others+Skin+Ear | 0.76 | 0.62 | 0.27 | 2.19 |
|  | Constant | 3.19 | 0.00 | 1.79 | 5.66 |

#-Reference group; *-Significant at 90 % Confidence Interval
