# Supplementary material for: Hospital utilization and out of pocket expenditure in public and private sectors under the universal government health insurance scheme in Chhattisgarh State, India: Lessons for universal health coverage
Source: PLoS One. 2017 Nov 17;12(11):e0187904. doi: 10.1371/journal.pone.0187904 (PMC5693461; doi:10.1371/journal.pone.0187904)
Supplement: S3 Table — (DOCX) [file pone.0187904.s003.docx]

**S3 Table: Adjusted Odds Ratio of OOPE (medical expenses minus reimbursements) by characteristics and its 95% CI (N=856*)**

| **Characteristic** | | **Adjusted Odds Ratio** | **P value** | **95 % Confidence Interval** | |
| --- | --- | --- | --- | --- | --- |
|  |  |  |  | **Lower Limit** | **Upper Limit** |
| Gender | Men# | 1 |  |  |  |
|  | Women | 1.700 | 0.045 | 1.012 | 2.858 |
| Residence | Rural# | 1 |  |  |  |
|  | Urban | 0.740 | 0.202 | 0.466 | 1.175 |
| Social Group | ST# | 1 |  |  |  |
|  | SC | 0.807 | 0.531 | 0.413 | 1.577 |
|  | OBC | 0.812 | 0.398 | 0.502 | 1.316 |
|  | Others | 2.329 | 0.066 | 0.947 | 5.732 |
| UMPCE | Q1# | 1 |  |  |  |
|  | Q2 | 0.618 | 0.125 | 0.334 | 1.143 |
|  | Q3 | 1.293 | 0.412 | 0.700 | 2.388 |
|  | Q4 | 1.471 | 0.240 | 0.773 | 2.799 |
|  | Q5 | 1.532 | 0.259 | 0.730 | 3.213 |
| Insurance | No Insurance# | 1 |  |  |  |
|  | Government Insurance | 0.265 | 0.000 | 0.174 | 0.405 |
| Type of Ailment | Infection# | 1 |  |  |  |
|  | Cancers* |  |  |  |  |
|  | Blood Diseases+ Endocrine Metabolic, Nutritional | 0.659 | 0.386 | 0.256 | 1.694 |
|  | Psychiatric & Neurological | 1.302 | 0.693 | 0.350 | 4.838 |
|  | Eye | 0.467 | 0.131 | 0.173 | 1.255 |
|  | Cardio-Vascular | 0.936 | 0.913 | 0.289 | 3.037 |
|  | Respiratory | 2.352 | 0.440 | 0.268 | 20.604 |
|  | Gastro-Intestinal | 1.338 | 0.562 | 0.500 | 3.584 |
|  | Musculo-Skeletal+ Genito-Urinary | 1.935 | 0.265 | 0.606 | 6.172 |
|  | Obstetric+ Childbirth | 0.516 | 0.024 | 0.290 | 0.918 |
|  | Injuries | 1.601 | 0.244 | 0.726 | 3.529 |
|  | Others+Skin+Ear | 0.663 | 0.513 | 0.193 | 2.274 |
| Level of hospital | Private hospital# | 1 |  |  |  |
|  | HSC/ANM/ASHA/AWW | 0.088 | 0.000 | 0.023 | 0.344 |
|  | PHC/dispensary/CHC/mobile medical unit | 0.092 | 0.000 | 0.041 | 0.211 |
|  | Public hospital | 0.146 | 0.000 | 0.085 | 0.251 |

#Reference Group *Cancer cases had all incurred OOP expenses and therefore cell frequency is zero
